# Death and Medical Power: An Ethical Analysis of Dutch Euthanasia Practice

**DOI:** 10.1038/sj.bjc.6603373

**Published:** 2006-10-17

**Authors:** D Oliver

**Affiliations:** 1 University of Kent, Kent, UK; 2 Wisdom Hospice, Rochester, UK

This interesting book is written by two Dutch ethicists, although neither is now working in the Netherlands. It is well researched with excellent information on the various legal cases that have been instrumental in the development of the euthanasia legislation and is well referenced.

Any publication looking at this difficult subject area involves consideration of complex issues, where there are strongly held arguments on both sides. This book argues against the development of medically procured death (MPD) – including both euthanasia and physician-assisted suicide.

There is discussion of the development of the rationale for MPD, which they argue came from concerns within society about the over intervention in the terminal phases of disease and abandonment by the medical profession of the dying. Within the Netherlands, a country with liberal views and a culture of tolerance, there was increased consideration of the ending of life – with the aim of ending a life that was ‘unbearable’. This arose as society became more secularised with increased emphasis on care at home through general practice and the increase in the anthropological approach to medical care. Initially, doctors who ended a patient's life at their request were prosecuted but received only a suspended sentence or no conviction at all. These cases are discussed in detail leading up to the change in the law in 2001 in the Netherlands – legalising euthanasia-stating the requirements of care to which physicians must abide to avoid prosecution and delineates the reporting procedures.

The prime aim of this book seems to be to discuss the development of the legal changes and expose the flaws that the authors see in the arguments – both legal and ethical – supporting the use of MPD. They suggest that one of the prime arguments for MPD is patient autonomy. However, this raises serious concerns as to how patients make these decisions

- Are they given sufficient and clear information?
- Are some physicians suggesting MPD as a way of coping with unbearable symptoms?
- Is there a progression to non-voluntary termination of life, when patients are killed without their competent request, such as for infants and demented?

Moreover, as not all requests for euthanasia are complied with – only about one-third of request result in assisted death – it would seem that patient autonomy is not always paramount. In fact, the authors argue that the physician becomes even more powerful and is put in the position of passing judgement on a patient's life and quality of life. Thus, in response to the medicalisation of death and the abandonment of the dying, physicians are now medicalising the decisions about MPD. Autonomy is not always respected, although this is used by many of the protagonists as a justification for MPD.

This book argues cogently against MPD and presents good evidence for this position. Although there is little counterargument presented for someone wanting to consider the ethical issues involved, this book will provide much food for thought.

